# 

**Published:** 1875-06

**Authors:** 


					﻿Vol. 22.
JUNE, 1875.
No. 6.
TWENTY-SECOND YEAR
HALL’S
Journal of Health.
PUBLISHED BY THE AMERICAN AND FOREIGN
PUBLICATION CO., 137 EIGHTH ST,, NEW YORK.
Agents for the Trade t
AMERICAN NEWS COMPANY, 121 NASSAU STREET.
NEW YORK NEWS COMPANY, 18 BEEKMAN STREET.
Terms, Two Dollars a Tear.
SINGI.E NUMBER, 20 CENTS.
				

